# A circular RNA, circSMARCA5, inhibits prostate cancer proliferative, migrative, and invasive capabilities via the miR-181b-5p/miR-17-3p-TIMP3 axis

**DOI:** 10.18632/aging.203408

**Published:** 2021-08-13

**Authors:** Xin Xie, Fu-Kang Sun, Xin Huang, Cheng-He Wang, Jun Dai, Ju-Ping Zhao, Chen Fang, Wei He

**Affiliations:** 1 Department of Urology, Ruijin Hospital, Shanghai Jiao Tong University School of Medicine, Shanghai 200025, P.R. China

**Keywords:** prostate cancer, circSMARCA5, miR-17-3p, miR-181b-5p, TIMP3

## Abstract

SMARCA5 (circSMARCA5) is involved in the occurrence of different cancers, but its role in prostate cancer carcinogenesis and metastatic transformation remains elusive. Thus, we evaluated the circSMARCA5 functional relevance in prostate cancer and its associated molecular mechanism. First, circSMARCA5 expression and function in this cancer were evaluated. To determine the miR-181b-5p/miR-17-3p target and clarify how circSMARCA5 regulates the miR-181b-5p-TIMP3 and miR-17-3p-TIMP3 axis, RNA immunoprecipitation, biotin-coupled microRNA capture, luciferase reporter, Western blot, and quantitative real-time PCR assays were employed. In primary and metastatic prostate cancer tissues, circSMARCA5 was significantly downregulated compared with normal controls. Functionally, circSMARCA5 exhibited a suppressive effect on prostate cancer cells' metastasis and growth. At the molecular level, circSMARCA5 could affect the tissue inhibitor of metalloproteinases 3 (TIMP3) expression through miR-181b-5p or miR-17-3p interactions. Moreover, lysine acetyltransferase 5 (KAT5) induced circSMARCA5 biogenesis and regulated the miR-181b-5p-TIMP3 and miR-17-3p-TIMP3 axis. These results suggested that targeting circSMARCA5-miR-181b-5p-TIMP3 and circSMARCA5-miR-17-3p-TIMP3 axis might be a novel therapeutic strategy for prostate cancer.

## INTRODUCTION

Prostate cancer is a common male cancer with increasing morbidity and mortality rates in China [1, 2]. Despite recent progress in prostate cancer patient's five-year survival rate, it remains a significant medical challenge. Particularly, resistance to radiotherapy, chemotherapy, or androgen deprivation therapy can lead to tumor metastasis and recurrence [3]. Prostate cancer pathogenesis and development are complicated and have different deregulated oncogenes and tumor-suppressors involved [4]. Therefore, elucidate prostate cancer genetic mechanisms to understand its metastatic capability and improve clinical therapy efficiency is critical.

Circular RNAs (circRNAs) are not translated into proteins [5, 6], and their aberrant expression has been suggested to promote, or suppress, tumorigenesis, including growth, metastasis, and progression [7]. The SMARCA5 gene - which encodes the circRNA SMARCA5 (circSMARCA5) - is located at chr4:144464662-144465125. Accumulating evidence suggests that circSMARCA5 is implicated in several cancer types, including glioblastoma multiforme, gastric cancer, hepatocellular carcinoma, and prostate cancer [8–10]. However, how circSMARCA5 is involved in prostate cancer metastatic transformation remains unknown.

MicroRNAs (miRNAs) are involved in many human malignancies and have been considered a therapeutic target due to their roles in the regulation of tumor cell behaviors [11]. circRNAs could sponge miRNA and control their expression, subsequently affecting their binding to mRNAs targets [12]. For instance, miR-181b-5p and miR-17-3p oncogenic roles in tumors proliferative, invasive, and metastatic capabilities were previously reported [13, 14]. Additionally, circSMARCA5 regulatory effect on miR-181b-5p and miR-17-3p in HCC was previously reported [10]. However, if circSMARCA5 regulates prostate cancer cells’ behaviors via miR-181b-5p and/or miR-17-3p sponging remains unknown. Therefore, we evaluated circSMARCA5 expression, role, and mechanism in the regulation of prostate cancer cells’ proliferative and invasive capabilities.

## RESULTS

### Low circSMARCA5 expression in metastatic prostate tumors

**Figure 1:**
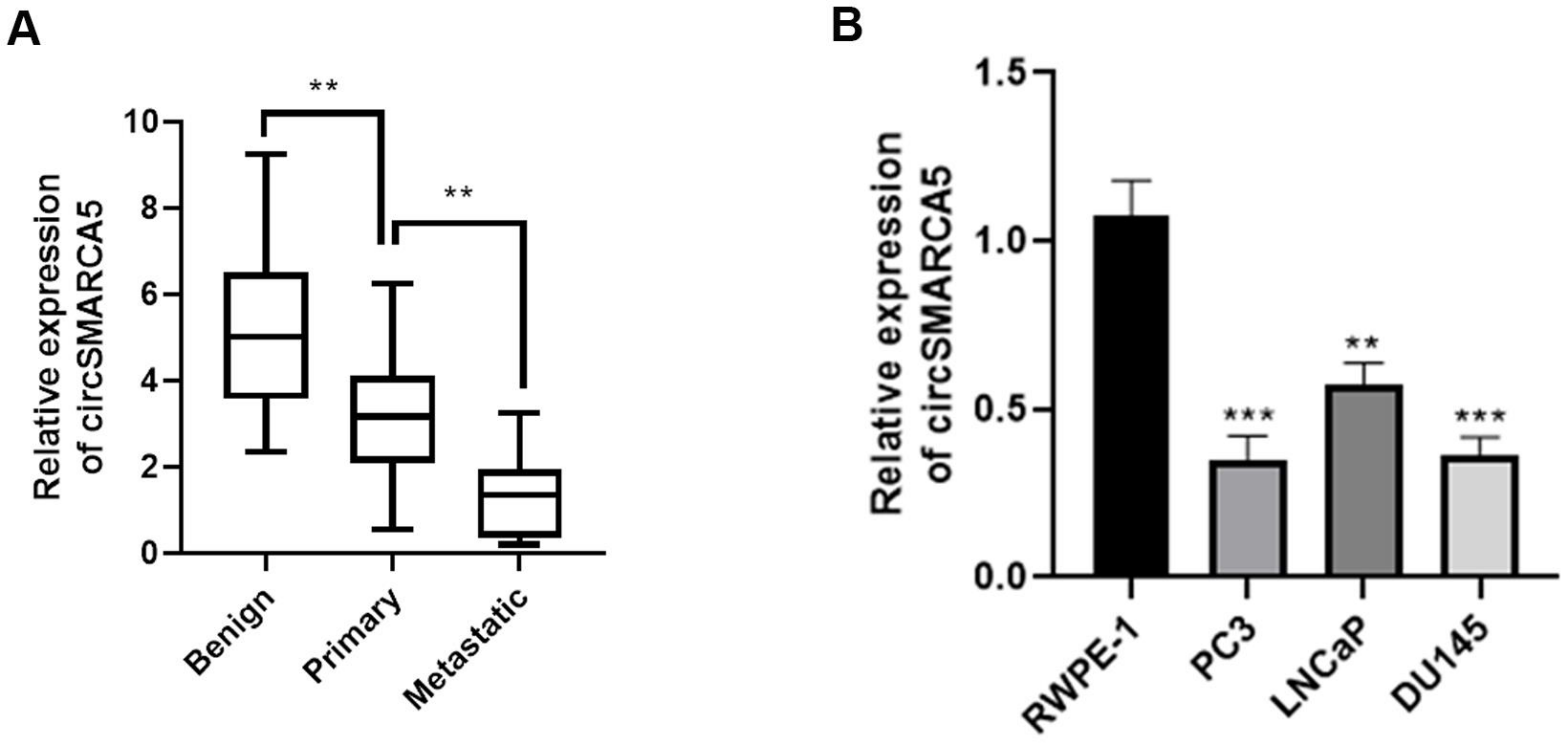
**circSMARCA5 expression in prostate tumors.** (**A**) The circSMARCA5 expression pattern in prostate cancer samples and benign controls was determined by qRT-PCR. (**B**) circSMARCA5 levels were measured in RWPE-1, DU145, LNCaP, and PC3 cells. *, **, *** represent *p* ≤ 0.05, *p* ≤ 0.01, *p* ≤ 0.001, respectively. Assays were performed at least three times.

First, we detected the circSMARCA5 (has_circ_0001445, located in chr4:144464661-144465125) expression pattern in prostate cancer. Primers that target and amplify the circSMARCA5 back-splice site were designed to detect its expression in prostate cancer. Malignant tumor tissues had significantly low circSMARCA5 expression, compared with primary samples, which was also observed in metastatic samples (Figure 1A). Additionally, we observed lower circSMARCA5 expression in DU145, LNCaP, and PC3 cells, compared to RWPE-1 cells (Figure 1B). Collectively, these data indicated that circSMARCA5 expression is involved in prostate malignant behaviors.

### circSMARCA5 inhibits prostate tumors proliferative, migrative, and invasive capabilities *in vitro*

**Figure 2:**
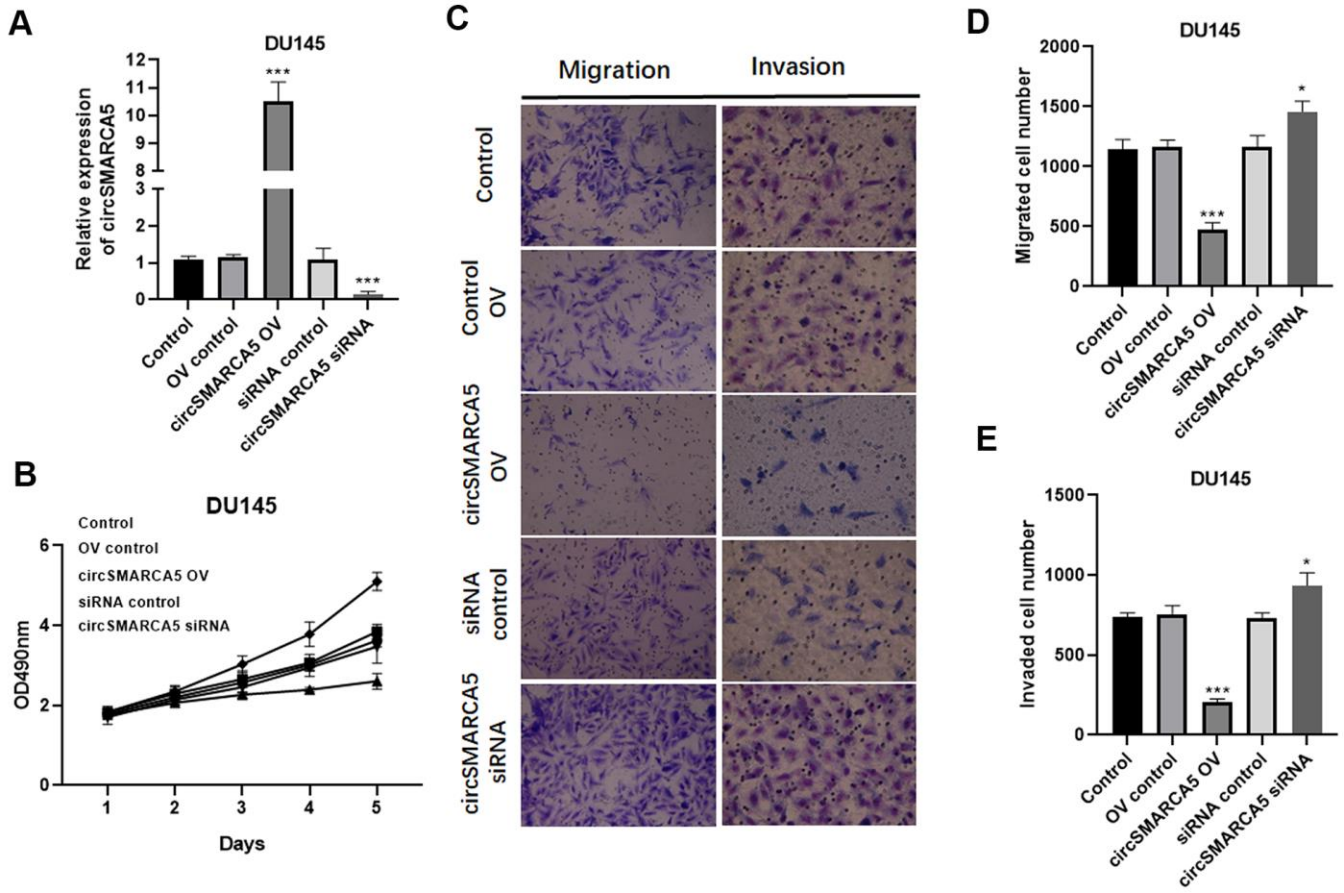
**circSMARCA5 inhibits DU145 cell proliferative, migrative, and invasive capabilities.** circSMARCA5 was upregulated or downregulated in DU145 cells. (**A**) circSMARCA5 expression in the cells with indicated treatments. (**B**) DU145 cells’ growth with different treatments. The Transwell assay results showed the migration (**C**, **D**) and migration (**C**, **E**) of DU145 cells after indicated treatment. Magnification ×100. *, **, *** represent *p* ≤ 0.05, *p* ≤ 0.01, *p* ≤ 0.001, respectively. Assays were performed at least three times.

**Figure 3:**
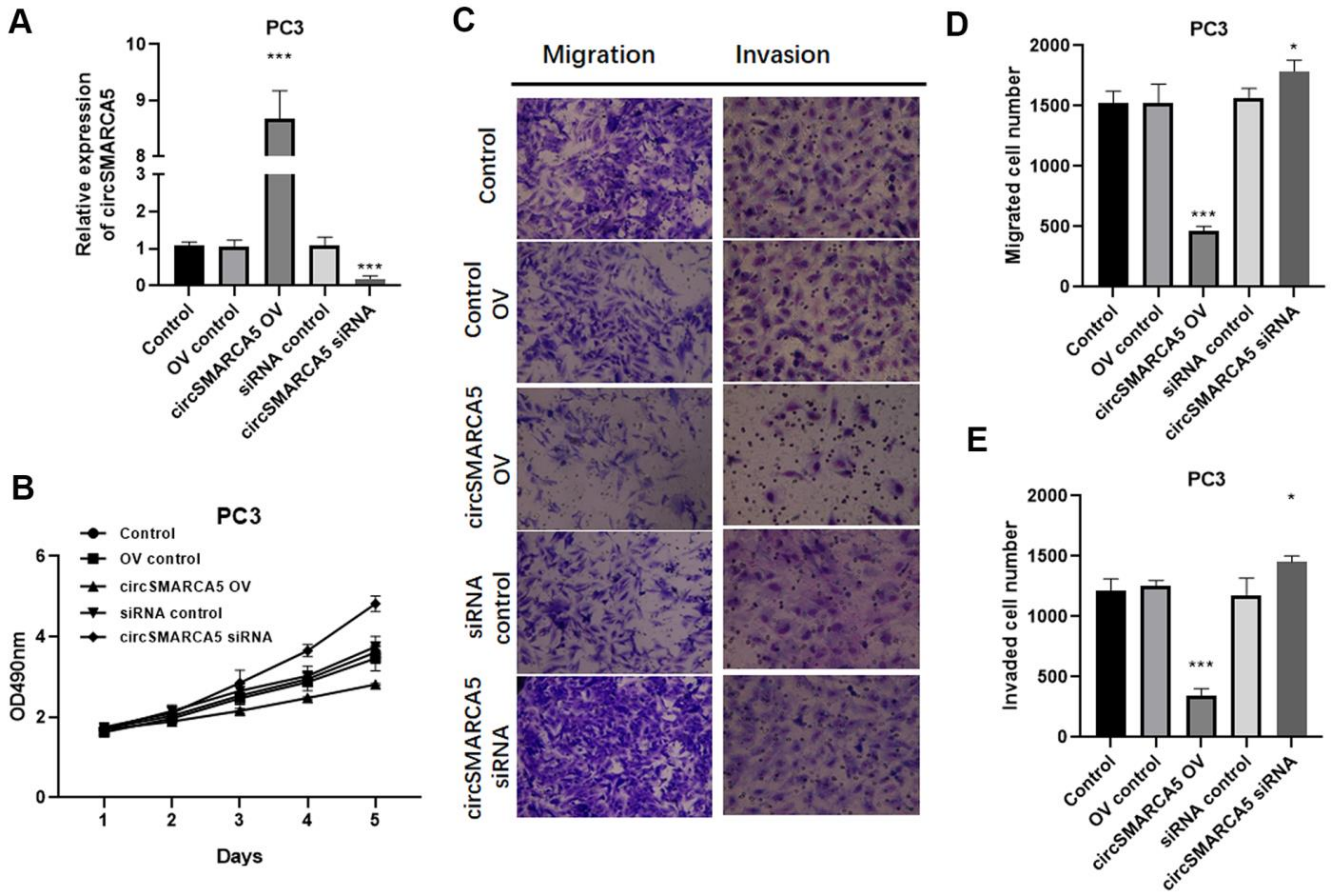
**circSMARCA5 inhibits PC3 cell proliferative, migrative, and invasive capabilities.** circSMARCA5 was upregulated or downregulated in PC3 cells. (**A**) The qRT-PCR results showed circSMARCA5 level in PC3 cells with indicated treatment. (**B**) PC3 cells’ growth with different treatments. The Transwell assay results showed the migration (**C**, **D**) and migration (**C**, **E**) of PC3 cells after indicated treatment. Magnification ×100. *, **, *** represent *p* ≤ 0.05, *p* ≤ 0.01, *p* ≤ 0.001, respectively. Assays were performed at least three times.

To further reveal circSMARCA5 functional relevance in prostate carcinogenesis and metastatic transformation, tumor cellular behaviors in which circSMARCA5 expression was impaired or enhanced were examined. circSMARCA5 was successfully overexpressed in DU145 cells (Figure 2A). Accordingly, circSMARCA5 upregulation suppressed cell growth, migration, and invasion (Figure 2B–2E). DU145 cells with circSMARCA5 knockdown exhibited the opposite effect on these processes (Figure 2B–2E). Similar effects were detected in PC3 cells (Figure 3A–3E). Altogether, these data validated that circSMARCA5 has a tumor-suppressive effect in prostate cancer.

### circSMARCA5 inhibits tumor progression and metastasis *in vivo*

**Figure 4:**
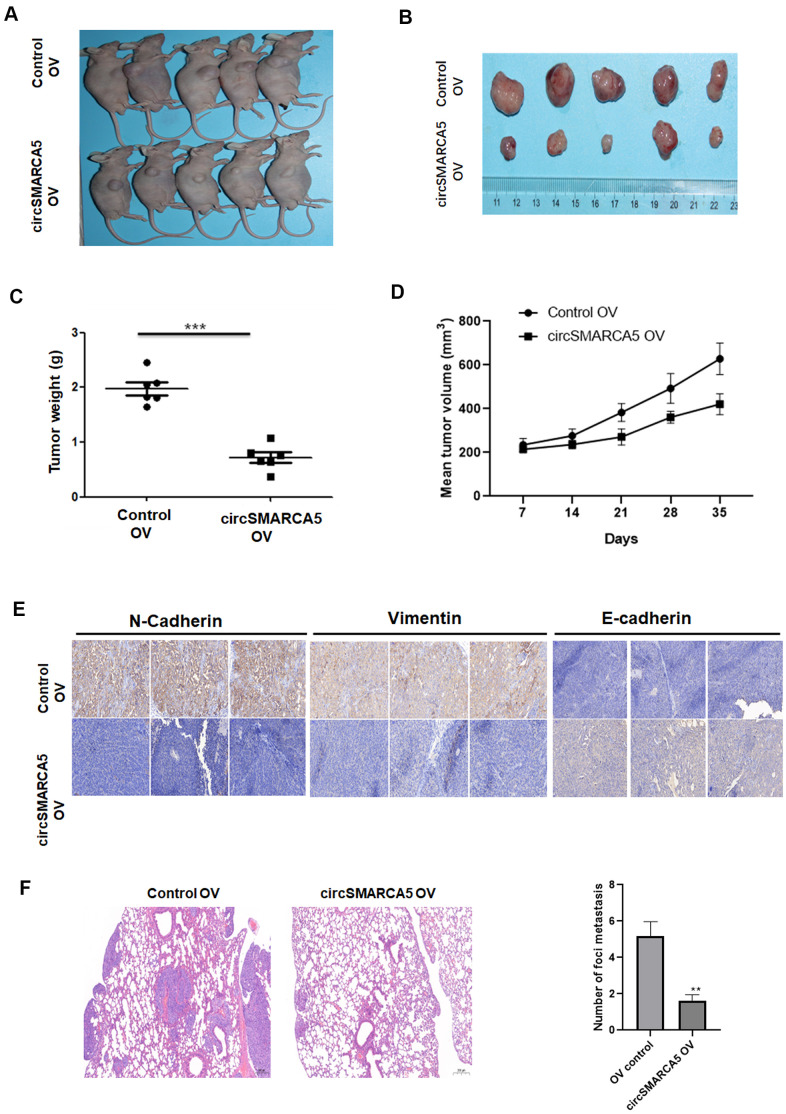
**circSMARCA5 suppresses tumor proliferative and invasive potential *in vivo*.** Wild-type and circSMARCA5-overexpressed DU145 cells were injected into nude recipient mice. Every week, information about tumor size was collected. The tumor tissues were removed and the weight was determined four weeks after injection. Representative mice and resected tumors images (**A**, **B**). (**C**, **D**) Tumor weight and volume. (**E**) IHC staining of EMT markers, including E-cadherin, vimentin, and N-cadherin. Magnification ×20. (**F**) H&E staining and lung microscopic nodules quantification of each group. Magnification ×20. *, **, *** represent *p* ≤ 0.05, *p* ≤ 0.01, *p* ≤ 0.001, respectively.

Next, we evaluated the circSMARCA5 role using a prostate cancer xenograft mouse model. circSMARCA5 overexpression in DU145 cells strongly suppressed tumor growth (Figure 4A–4D). Immunohistochemistry staining showed that overexpressed circSMARCA5 decreased vimentin and N-cadherin levels, and enhanced E-cadherin levels (Figure 4E). Moreover, a lung metastasis model confirmed that SMARCA5 upregulation dramatically inhibited lung metastasis (Figure 4F). Overall, these results suggested that circSMARCA5 overexpression can inhibit prostate tumor growth and metastasis.

### circSMARCA5 sponged miR-181b-5p/miR-17-3p to regulate TIMP3

**Figure 5:**
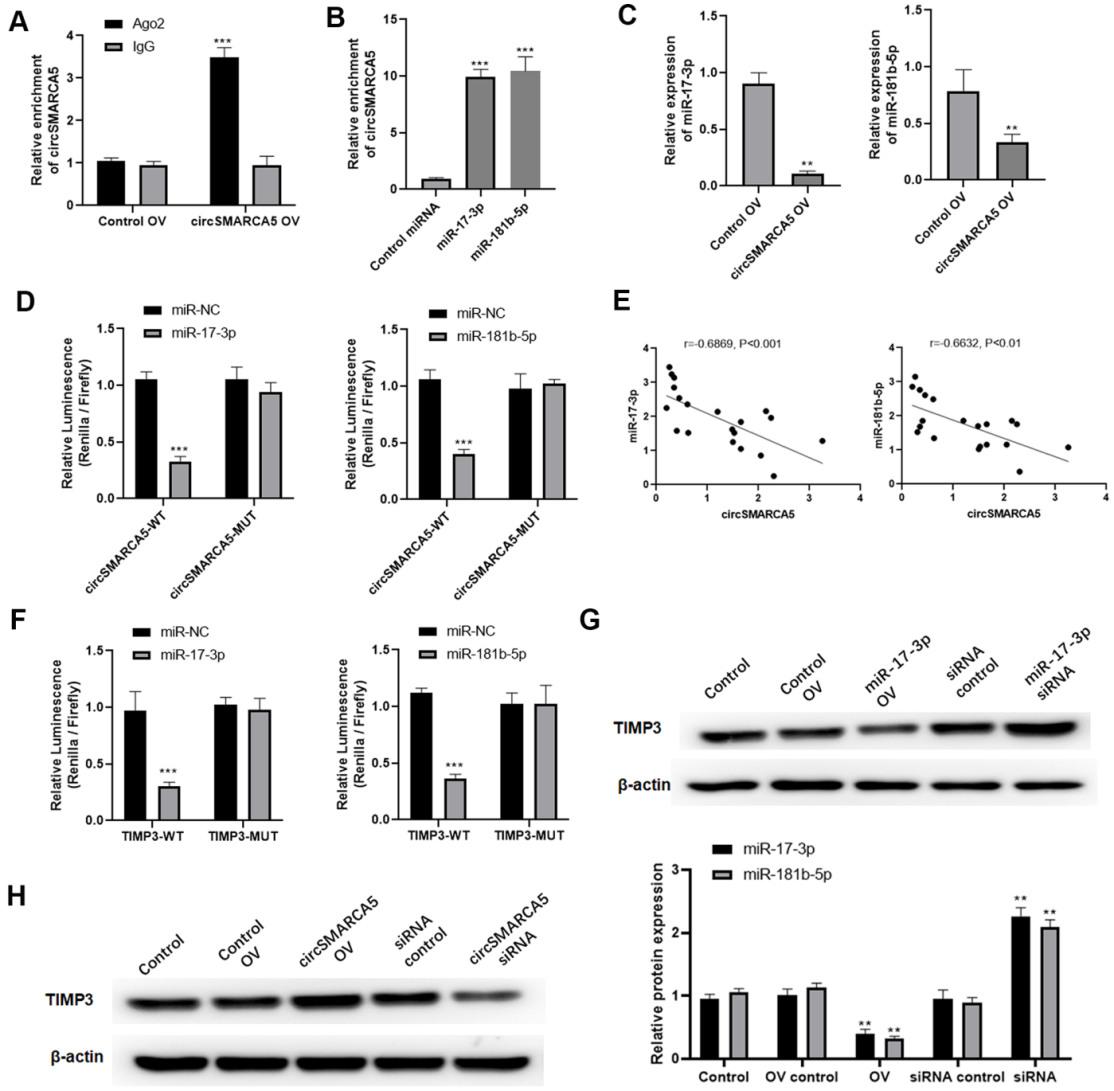
**circSMARCA5 sponged miR-181b-5p/miR-17-3p to regulate TIMP3.** (**A**) qRT-PCR for the pulled-down circSMARCA5. (**B**) qRT-PCR for circSMARCA5 enrichment in DU145 cell lysates. (**C**) qRT-PCR for miR-181b-5p and miR-17-3p. (**D**) Luciferase activities in the cells with indicated treatments. (**E**) Correlation between circSMARCA5 expression and miR-181b-5p, or miR-17-3p, in prostate tumor tissues. (**F**) Luciferase activities in the cells with indicated treatments. (**G**) Western blot for TIMP3 expression after overexpression or downregulation of miR-17-3p. (**H**) Western blot for the expression of TIMP3 after circSMARCA5 overexpression, or downregulation. *, **, *** represent *p* ≤ 0.05, *p* ≤ 0.01, *p* ≤ 0.001, respectively. Assays were performed at least three times.

Then, we explored if circSMARCA5 could bind to miRNAs. circSMARCA5 was specifically enriched by Ago2 antibody but not IgG (Figure 5A), suggesting that it may interact with Ago2 and miRNAs. Additionally, miR-181b-5p and miR-17-3p were sponged by circSMARCA5 [10]. Our results revealed that circSMARCA5 sponge not only miR-181b-5p but also miR-17-3p, compared to the control (Figure 5B). Meanwhile, circSMARCA5 upregulation dramatically suppressed miR-181b-5p and miR-17-3p expressions in DU145 cells (Figure 5C). Also, compared to control cells, cells transfected with miR-181b-5p and pGL3-circSMARCA5-WT, or miR-17-3p mimic and pGL3-circSMARCA5-WT together, had a very low luciferase activity (Figure 5D). Using clinical samples, negative correlations were found between miR-181b-5p and circSMARCA5, as well as miR-17-3p and circSMARCA5 (Figure 5E). Next, DU145 cells transfected with miR-181b-5p mimic and pGL3-TIMP3-WT, or miR-17-3p mimic and pGL3-TIMP3-WT showed significant low luciferase values, indicating that TIMP3 can be specifically targeted by miR-181b-5p and miR-17-3p (Figure 5F). Moreover, overexpression and knockdown assays demonstrated that TIMP3 expression was negatively regulated by miR-181b-5p and miR-17-3p (Figure 5G). Then, to explore the interaction between circSMARCA5/miRNA/TIMP3, circSMARCA5 was upregulated or downregulated. Consequently, circSMARCA5 overexpression increased TIMP3 protein level in DU145 cells, whereas the downregulation suppressed TIMP3 levels (Figure 5H). Altogether, these results suggested that circSMARCA5 regulates TIMP3 expression through miR-181b-5p/miR-17-3p sponging.

### circSMARCA5 suppresses growth and metastasis via the miR-181b-5p/miR-17-3p-TIMP3 axis

**Figure 6:**
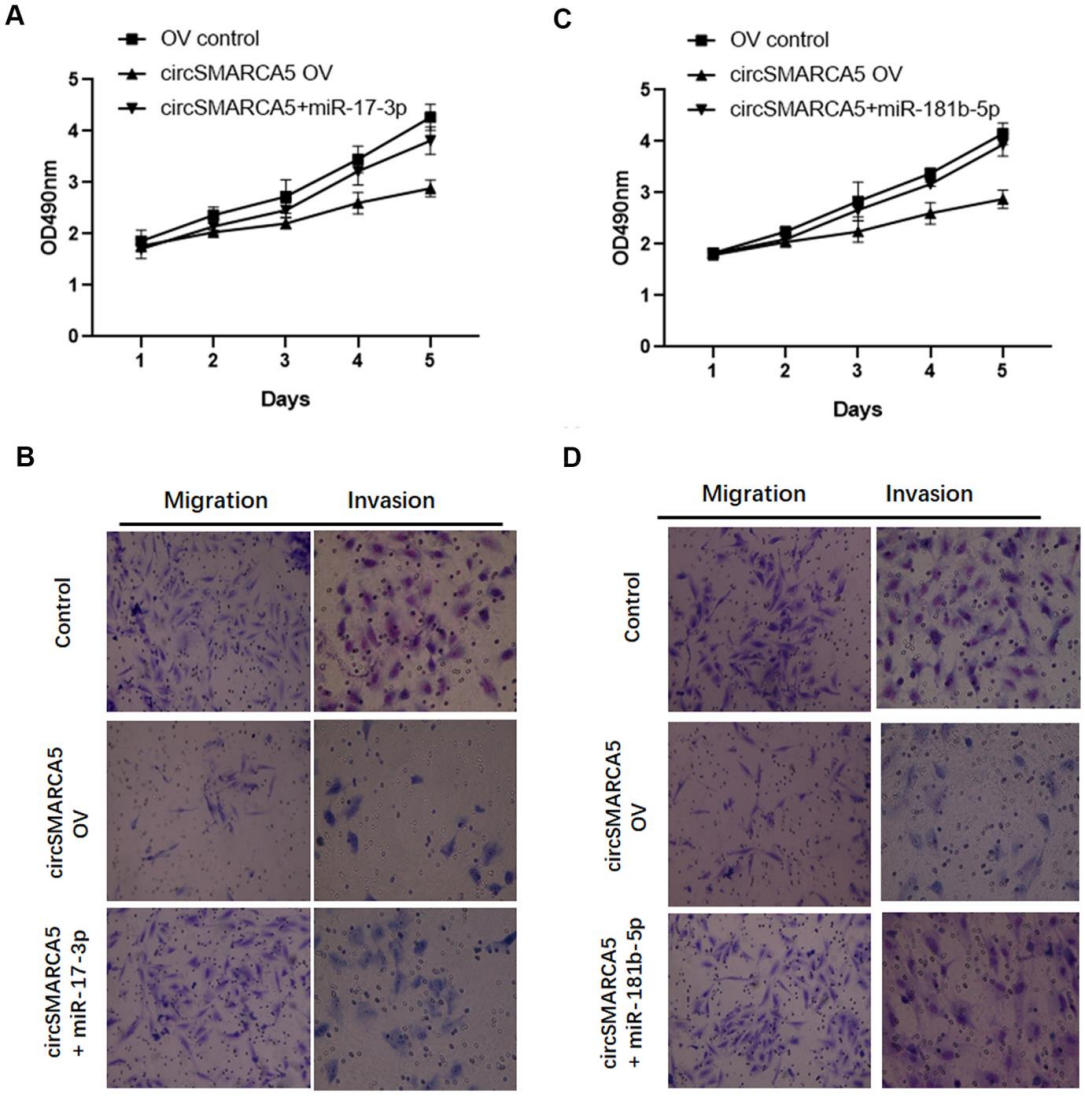
**circSMARCA5 suppresses proliferative and invasive potential via the miR-181b-5p/miR-17-3p-TIMP3 axis.** (**A**, **B**) circSMARCA5 overexpressing vectors were transfected into DU145 cells with and without miR-17-3p mimic. Cell proliferation, migration, and invasion were determined. (**C**, **D**) circSMARCA5 overexpressing vectors were transfected into DU145 cells with and without miR-181b-5p mimic. Cell proliferation, migration, and invasion were measured. *, **, *** represent *p* ≤ 0.05, *p* ≤ 0.01, *p* ≤ 0.001, respectively. Assays were performed at least three times.

Based on the results presented above, we speculated that circSMARCA5 could play tumor-suppressive effects via miR-181b-5p-regulated and/or miR-17-3p-regulated TIMP3. To validate this hypothesis, we transfected circSMARCA5 and miR-181b-5p mimic or circSMARCA5 and miR-17-3p mimic together into DU145 cells. Consequently, the tumor-inhibitory effects of circSMARCA5 overexpression were abolished by exogenous miR-17-3p addition (Figure 6A, 6B). Consistently, miR-181b-5p overexpression also attenuated the circSMARCA5 anti-tumor effects (Figure 6C, 6D). These results demonstrated that circSMARCA5 exerts tumor-suppressive effects by miR-181b-5p/miR-17-3p regulation.

### Lysine acetyltransferase 5 (KAT5) mediates circSMARCA5 biogenesis

**Figure 7:**
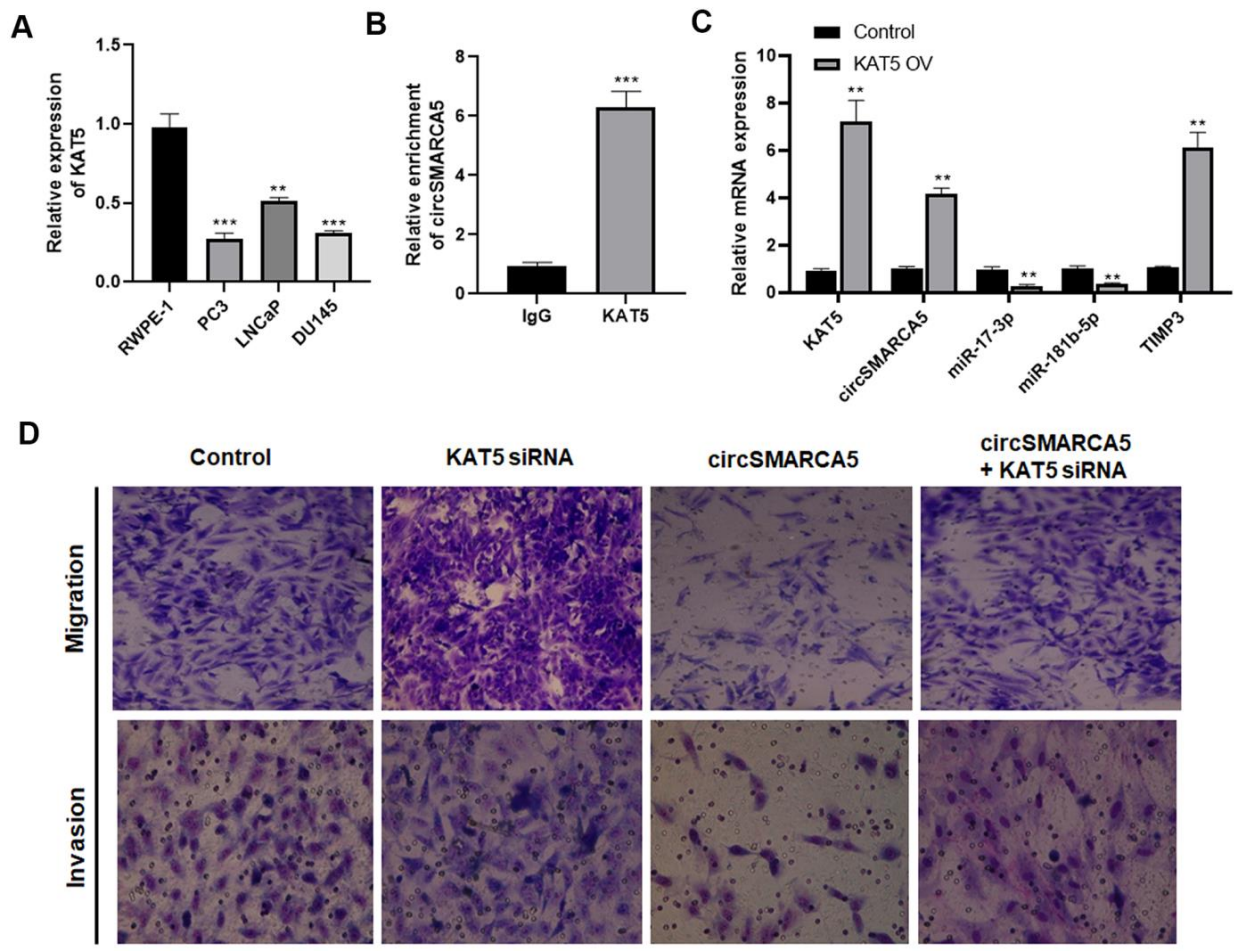
**KAT5 induces circSMARCA5 production.** (**A**) The KAT5 expression pattern in RWPE-1, DU145, LNCaP, and PC3 cells. (**B**) Detection of pulled-down circSMARCA5 by qRT-PCR. (**C**) qRT-PCR of KAT5, miR-181b-5p, TIMP3, and miR-17-3p in DU145 cells with and without KAT5 overexpression. (**D**) U145 cells were transfected with circSMARCA5-expressing vector and KAT5-specific siRNA, alone or in combination. Migration and invasion were determined by Transwell assays. *, **, *** represent *p* ≤ 0.05, *p* ≤ 0.01, *p* ≤ 0.001, respectively. Assays were performed at least three times.

Furthermore, the mechanism underlying the circSMARCA5 decrease in prostate cancer cells was explored. Our previous study focused on KAT5's effects on prostate cancer pathogenesis [15]. Currently, compared to the control prostate epithelial cells RWPE-1, the KAT5 level was significantly decreased in DU145, LNCaP, and PC3 cells (Figure 7A). To explore the interaction between KAT5 and circSMARCA5 formation, RIP assays with a KAT5 antibody revealed significant circSMARCA5 enrichment in the anti-KAT5 immunoprecipitants, compared to those in IgG controls (Figure 7B). Next, we determined if KAT5 could regulate the circSMARCA5 biogenesis. Results from qRT-PCR showed that KAT5 increased the circSMARCA5 production (Figure 7C), suggesting that KAT5 binds to circSMARCA5 and induces its biogenesis.

To evaluate the KAT5 effect on circSMARCA5-regulated cellular behaviors, DU145 cells were transfected with circSMARCA5-expressing vector, KAT5 siRNA, or their negative controls. As a result, KAT5 silencing promoted migrative and invasive capabilities and restored their inhibition induced by circSMARCA5 overexpression (Figure 7D). Collectively, these results suggested that KAT5-mediated circSMARCA5 biogenesis affects the circSMARCA5-miR-181b-5p-TIMP3 and/or circSMARCA5-miR-17-3p-TIMP3 axis in prostate cancer cells.

## DISCUSSION

circSMARCA5 is implicated in the tumorigenesis of different cancers [9, 10]. However, the circSMARCA5 biological relevance in prostate carcinogenesis and metastatic transformation was unknown. In this study, we demonstrated that the circSMARCA5-miR-181b-5p/ miR-17-3p-TIMP3 axis is critical in prostate cancer development.

The circSMARCA5 tumor-suppressive role, discovered in the present study, was consistent with previous studies. For example, Davide et al. found that circSMARCA5 was negatively correlated with angiogenesis in glioblastoma multiforme [16]. Similarly, a negative correlation between circSMARCA5 and clinicopathological features, including cancer diameter, invasion, and the tumor-node-metastasis stage were observed in HCC [17]. These studies suggested that circSMARCA5 may act as a prognostic marker for cancers. Furthermore, functional assays revealed that circSMARCA5 inhibits the migrative and invasive capabilities in some tumors, such as cervical cancer, HCC, and lung cancer [18, 19]. Both tumor suppressive and promotive functions of circSMARCA5 were observed in prostate cancer [10, 20]. Our present results revealed that circSMARCA5 was negatively associated with prostate cancer proliferative, migrative, and invasive capabilities. Consistently, our *in vivo* assay also confirmed that circSMARCA5 exhibited a suppressive role in prostate tumors growth and metastasis.

circRNAs can play diverse roles such as RNA-binding proteins, miRNA sponges, and transcriptional regulators [21]. For instance, circ-Sirt1 can sponge the miR-132/212 cluster to alleviate their suppressive effects on SIRT1 expression [22]. circSMARCA5 sponges the miR-19b-3p, leading to invasion, migration, and growth inhibition in NSCLC cells [14]. Through bioinformatics analysis, we predicted the interacting miRNA and its regulated target gene. Consequently, miR-181b-5p and miR-17-3p had conserved circSMARCA5 binding sites and were shown to be onco-miRNAs with proliferative, invasive, and metastatic potential in tumor cells [13, 14]. Additional results confirmed that circSMARCA5 could sponge miR-181b-5p and miR-17-3p. TIMP3 is a tumor-suppressive factor that if lost will accelerates tumor invasion and metastasis in prostate cancer [23]. Thus, we further identified that TIMP3 can be targeted by miR-181b-5p and miR-17-3p, implying a regulatory role of the circSMARCA5-miR-181b-5p-TIMP3 and/or circSMARCA5-miR-17-3p-TIMP3 axis involved in prostate cancer progression. Indeed, we found that miR-181b-5p or miR-17-3p attenuated the circSMARCA5 inhibitory role on tumor cell migration and invasion, validating that circSMARCA5 could sponge miR-181b-5p, as well as miR-17-3p, to promote TIMP3 and inhibit malignant behaviors in prostate cancer.

Furthermore, the mechanism regarding the regulation of circSMARCA5 deregulation in prostate cancer remains unknown. As a main NuA4 subunit, KAT5 can initiate target genes expression and regulate histone acylation [24]. KAT5 can induce prostate cancer cells’ apoptosis and suppress their growth, representing a gene therapy target [25]. Furthermore, KAT5 can directly interact with the circRNA, circRHOT1, which is involved in hepatocellular carcinoma progression [26]. The pathogenic effect of KAT5 in prostate cancer was observed in our previous study [16]. Herein, decreased KAT5 expression in prostate cancer tissues was observed and KAT5 upregulation promoted circSMARCA5 production. Functionally, KAT5 suppression enhanced the migrative and invasive capabilities and reversed the circSMARCA5 suppressive effects, suggesting a novel link of the KAT5-mediated circSMARCA5 production in prostate cancer.

Therefore, KAT5-mediated circSMARCA5 biogenesis suppressed prostate cancer invasive, migrative, and progressive capabilities, *in vitro* and *in vivo*. Mechanically, circSMARCA5 plays a tumor-suppressive role via the miR-181b-5p-TIMP3 or miR-17-3p-TIMP3 axis in prostate cancer. These results suggested that the KAT5-circSMARCA5-miR-181b-5p-TIMP3 and/or KAT5-circSMARCA5-miR-17-3p-TIMP3 axis might represent a novel target for prostate cancer therapy.

## MATERIALS AND METHODS

### Patients, specimens, and cell culture

Patients (n = 20) who underwent radical prostatectomy for primary, metastatic, and benign prostate cancer, and prostate transurethral resected patients were enrolled in this study. The Ethics Committee of Shanghai Jiao Tong University approved all procedures. RPMI1640 medium was used to culture the DU145, PC3, and LNCaP cells. The K-SMF medium was used for RWPE-1 cells.

### Transfection

The circSMARCA5-containing vector, the miR-181b-5p mimic, or miR-17-3p mimic, KAT5 siRNA (5’-CAAGTGTCTTCAGCGTCATTT-3’), and their respective negative controls were transfected, or co-transfected, via lentivirus-mediated transfection system. Lipofectamine 2000 (Thermo Fisher, MA, USA) was employed in the lentiviruses package. Finally, viruses were purified, then infected the cells.

### Cell proliferation

First, prostate cancer cells were seeded (4 × 10^3^). Then, the tetrazolium compound (MTS; Promega, USA) was employed to determine cell growth. The values were determined at 490 nm after a 2-hour incubation at 37° C.

### Immunohistochemical analysis

After fixation with 10% PFA, tumor tissues were cut into 5 mm-thick sections. Slides were heated in a dry oven, deparaffinized, rehydrated through graded ethanol, and microwaved in 10 mM citric acid buffer (pH 6.0). Nonspecific binding was blocked with 10% goat serum and the primary antibodies against N-cadherin (#4061, CST, USA), vimentin (#5741, CST, USA), or E-cadherin (#3195, CST, USA) were incubated at 4° C overnight. Finally, the slides were incubated with horseradish peroxidase-labeled streptavidin for targeted proteins detection.

### Measurement of cell migrative and invasive capabilities

To evaluate migrative and invasive capabilities, a Transwell plate was employed. Cells were plated into the top part of the chambers. The Matrigel was used to coat the chambers to determine the cell’s invasive capability with indicated treatment. The normal medium was added to the bottom part of the Transwell plate. After 48-hour incubation, methanol was used to fix cells that migrated, or infiltrated, to the bottom of the champers. Finally, fixed cells were observed after crystal violet staining using a microscope.

### qRT-PCR

A qRT-PCR assay was performed using the SYBR Premix Ex TaqTM (TaKaRa, Otsu, Japan). The primers used were: cSMARCA5-F: GCTATCAAGCTCCATCCGCAT, cSMARCA5-R: TAAGACGAAGCACCGGA; TIMP3-F: TACCGAGGCTTCACCAAGA TGC, TIMP3-R: CATCTTGCCATCATAGACGCGAC; miR-17-3P-F: GCGACTGCAGTGA AGGCAC, miR-17-3p-R: AGTGCAGGGTCCGAGGTATT; miR-181b-5p-F: GCGAAC ATTCATTGC TGTCG, miR-181b-5p-R: AGTGCAGGGTCCGAGGTATT; KAT5-F: GGA ACTCACCACATTGCCTGTC, KAT5-R: CTCATTGCCTGGAGGATGTCGT; U6-F: CTC GCTTCGGCAGCACAT, U6-R: TTTGCGTGTCATCCTTGCG. GAPDH, or U6, was used as endogenous control.

### Western blot

The proteins were isolated from indicated lysed cells and 20 μg proteins were loaded into SDS-PAGE per lane. Protein bands were transferred onto polyvinylidene difluoride membranes. After washing and blocking three times, membranes were incubated with indicated primary and secondary antibodies against TIMP3 (ab39184, Abcam, USA). GAPDH (ab8245, Abcam, USA) was detected using HRP-conjugated secondary antibodies (Promega, USA). A chemiluminescence detection system (Millipore, WBKLS0500) was used to visualize the protein bands. The GAPDH (1:5000, Abcam, USA) was the normalization control.

### Dual-luciferase reporter assay

DU145 cells were transfected with wild (pGL3-circSMARCA5-WT and pGL3-TIMP3-WT), or mutant-type vectors (pGL3-circSMARCA5-MUT and pGL3-TIMP3-MUT) and miR-181b-5p or miR-17-3p mimics together. Then, after 48-hour incubation, the luciferase activity was measured.

### Animals

The Animal Ethics Committee of Shanghai Jiao Tong University reviewed and approved all procedures in this study. To induce the tumor xenograft murine model, 5 × 10^7^ DU145 cells were subcutaneously injected into nude mice. Additionally, mice were intraperitoneally injected with tumor cells for metastasis assessment. Tumors size was monitored once a week, and the formula length X width^2^ was employed to calculate its volume. For Hematoxylin and Eosin (H&E) staining, lung tissues were sectioned and fixed with 10% PFA and stained with H&E, based on standard protocols.

### RNA immunoprecipitation assay

The Biotin-labeled circSMARCA5 used in this study was synthesized by Sangon Biotech (Shanghai, China). After circSMARCA5 overexpression, cells were washed with PBS and fixed with 1% formaldehyde. Then, cells were incubated with streptavidin Dynabeads (Invitrogen, USA). The precipitated RNA was used to detect the targeted RNA by qRT-PCR. This experiment was repeated at least three times.

### Statistical analysis

All values are shown as means ± standard deviations (S.D.). The Student’s t-test was performed to determine the differences between indicated groups. A *p* < 0.05 was considered statistically significant.
